# Beyond the immediate tumor microenvironment: distinct T cell phenotype and function of tumor-adjacent healthy breast tissue associated with regional metastasis in breast cancer

**DOI:** 10.1186/2051-1426-2-S3-P269

**Published:** 2014-11-06

**Authors:** Yi Guo, Alfred Chan, Ingrid Cely, Jaime Shamonki, Michelle Kinnaird, Maggie DiNome, Begonya Comin-Anduix, Peter Sieling, Delphine Lee

**Affiliations:** 1 John Wayne Cancer Institute at Providence Saint John's Health Center, Santa Monica, CA, USA; 2 California Cryobank, CA, USA; 3 Department of Pathology, Providence Saint John's Health Center, Santa Monica, CA, USA; 4 David Geffen School of Medicine at UCLA, Los Angeles, CA, USA; 5 John Wayne Cancer Institute, Santa Monica, CA, USA

## 

While current breast cancer staging includes lymph node involvement as a key indicator of prognosis, little is known about how healthy tissue surrounding the immediate tumor microenvironment contributes to the loco-regional spread of disease. Previous studies of the role of the immune microenvironment in breast cancer have focused on the study of intratumoral T cells, reporting that increased intratumoral CD8+ T cell proportions with decreased CD4+ T cell proportions are associated with lymph node metastasis and rapid tumor progression. We hypothesized that T cells within the unaffected breast tissue ("healthy-adjacent" tissue) near the tumor (2-5 cm away from the tumor margin) may influence the local/regional spread of tumor cells. Since the amount of tissue available for study from the excision of primary tumors is limited, we utilized a previously established three-dimensional explant method to expand resident T cells from fresh breast cancer and healthy-adjacent breast tissue to investigate their phenotypic and functional qualities. We compared tissues from patients with LN metastasis (n = 8) and those without LN involvement (n = 7). We defined the surface phenotype of T cells derived from breast cancer and healthy-adjacent tissue using multiparameter flow cytometry. We found that healthy-adjacent tissue from patients with LN metastases exhibited a 1.6 fold lower percentage of CD4 T cells (p < 0.01) and 3.7 fold higher percentage of CD8 T cells (p < 0.05) than patients with no LN involvement. In contrast, we did not find any statistically significant differences in the number of intratumoral CD4 and CD8 T cells in patients with LN metastases versus those with no LN involvement. We hypothesized that qualitative T cell cytokine response from the surrounding healthy adjacent tissue may also be associated with LN metastases. Our preliminary data from the study of breast cancer patients (n = 6) showed some trends of increased Th2 cytokine and decreased pro-inflammatory cytokine associated with nodal metastases. These data suggest that the tissue extending beyond the tumor microenvironment (healthy-adjacent tissue) may contribute to immune surveillance and regional metastases. While this study size is small, it demonstrates the feasibility of using the explant method to interrogate healthy-appearing tumor adjacent tissue to investigate the surrounding breast tissue environment. Our preliminary findings also indicate the potential relevance of investigating the role of T cells in tumor adjacent healthy-appearing breast tissue to provide tumor immune surveillance. Further analysis of the T cells present in healthy adjacent breast tissue is warranted.

## Consent

Written informed consent was obtained from the patient for publication of this abstract and any accompanying images. A copy of the written consent is available for review by the Editor of this journal.

